# Assessment of Altmetrics and PlumX Metrics Scoring as Mechanisms to Evaluate the Top 100 Trending Hidradenitis Suppurativa Articles on Social Media: Cross-Sectional Study

**DOI:** 10.2196/23724

**Published:** 2020-11-19

**Authors:** Chapman Wei, Aaron Fong, Theodore Quan, Puneet Gupta, Adam Friedman

**Affiliations:** 1 George Washington University School of Medicine and Health Sciences Washington, DC United States; 2 Department of Dermatology George Washington University School of Medicine and Health Sciences Washington, DC United States

**Keywords:** altmetric, PlumX, social media, impact factor, hidradenitis suppurativa

## Abstract

**Background:**

Dermatologists are increasingly utilizing social media platforms to disseminate scientific information. New tools, such as altmetrics and PlumX metrics, have been made available to rapidly capture the level of scientific article dissemination across social media platforms. However, no studies have been performed to assess the level of scientific article dissemination across social media regarding hidradenitis suppurativa, a disease that is still currently not well understood.

**Objective:**

The aim of our study was to evaluate the utility of altmetrics and PlumX metrics by characterizing the top 100 “trending” hidradenitis suppurativa articles in the altmetric database by the altmetric attention score and PlumX score.

**Methods:**

Altmetric data components of the top 100 hidradenitis suppurativa articles were extracted from the altmetric database. Article citation count was found using Web of Science. PlumX field-weighted impact scores for each article were collected from the Scopus database. Journal title, open-access status, article type, and study design of original articles were assessed. Additionally, the altmetric attention score, PlumX score, and citation count were log transformed and adjusted by +1 for linear regression, and Spearman correlation coefficients were utilized to determine correlations.

**Results:**

Most of the top 100 “trending” hidradenitis suppurativa articles were published in *JAMA Dermatology* (n=27, 27%). The median altmetric attention score, PlumX score, and citation count were 25.5, 3.7, and 10.5, respectively. The most mentions regarding social media platforms came from Twitter. Although no correlation was observed between the citation count and altmetric attention score (*r^2^*=0.019, *P*=.17), positive correlation was observed between the citation count and PlumX score (*r^2^*=0.469, *P*<.001).

**Conclusions:**

Our research demonstrated that citation count is not correlated with the altmetric attention score, but is strongly correlated with the PlumX score regarding hidradenitis suppurativa articles at this point in time. With the continual increase of social media usage by medical professionals and researchers, this study can help investigators understand the best way to captivate their audience.

## Introduction

Dissemination of science-based peer-reviewed information is essential for increasing awareness of hidradenitis suppurativa, an inflammatory skin disease that is often underrecognized, leading to delayed diagnosis and higher severity at initial presentation [1]. Dermatologists are increasingly utilizing social media platforms to disseminate scientific information [2]. In contrast to medical journals, social media serves as a useful platform to inform the wider general public, both medical professionals and laypeople alike. New tools, including alternative metrics (altmetrics) and PlumX metrics, have been made available to evaluate the extent of an article’s dissemination across social media platforms [3,4]. Provided by altmetrics, the altmetric attention score is a weighted score of the amount of “online attention” a research article has received across social media platforms. These platforms include Facebook, Twitter, Google+, blogs, and others [5]. Similarly, PlumX has a PlumX field-weighted citation impact score (PlumX score) that is also a weighted score of the level of article dissemination across similar social media metrics and includes the number of citations, linkouts, and abstract views [6]. While the citation count or “impact factor” reflects the number of citations by other articles or journals, altmetrics and PlumX metrics reflect an article’s instantaneous attentiveness among news outlets, blogs, Twitter, Facebook, and other media platforms [7]. While these tools allow one to assess what kind of articles are garnering social media attention or “trending” on social media platforms, the assessment of the utility of altmetrics regarding hidradenitis suppurativa research has not been evaluated yet. Owing to the ability of these metrics to rapidly capture the level of scientific article dissemination, they have the potential of being used complementary with citation count and identifying high-impact hidradenitis suppurativa articles, since the traditional tool takes years to measure an article’s impact on field advancement. To evaluate the utility of these tools, we characterized the top 100 “trending” hidradenitis suppurativa articles across social media platforms captured in the altmetric database by altmetric attention scores complemented with PlumX scores and more established markers of manuscript value. Our study aimed to assess the social media platforms that most contribute to the dissemination of literature-based hidradenitis suppurativa information. We also sought to examine how alternative metrics compare with traditional metrics, such as citation count. We hypothesized that alternative metrics will be able to better complement more traditional metrics when evaluating an article’s quality.

## Methods

Utilizing Altmetric Explorer, we identified articles using the PubMed search criterion “hidradenitis suppurativa.” Altmetric data components of the top 100 altmetric attention score hidradenitis suppurativa articles were extracted in July 2020. The number of mentions from the following altmetric data components was extracted and examined: news mentions, blog mentions, policy mentions, Twitter mentions, Facebook mentions, Wikipedia mentions, Reddit mentions, Mendeley readers, and number of Dimension citations [6]. Mentions represent a measure of how many times a specific term, such as “hidradenitis suppurative,” has been referenced on social media channels. Dimension citations include grants, publications, citations, alternative metrics, clinical trials, and patents. Data were also collected about the journal impact factor, journal title, open-access status, where the articles were published, article type, article study design, and article citation count [4,5]. PlumX field-weighted citation impact scores for each article were collected from the Scopus database. Statistical analysis was performed using Prism 8 (GraphPad software), and statistical significance was defined as a *P* value <.05. Spearman correlation coefficients were utilized to determine correlations [2]. Altmetric attention scores, PlumX field-weighted citation impact scores, and citation count were log transformed and adjusted by +1 for linear regression.

## Results

**Figure 1:**
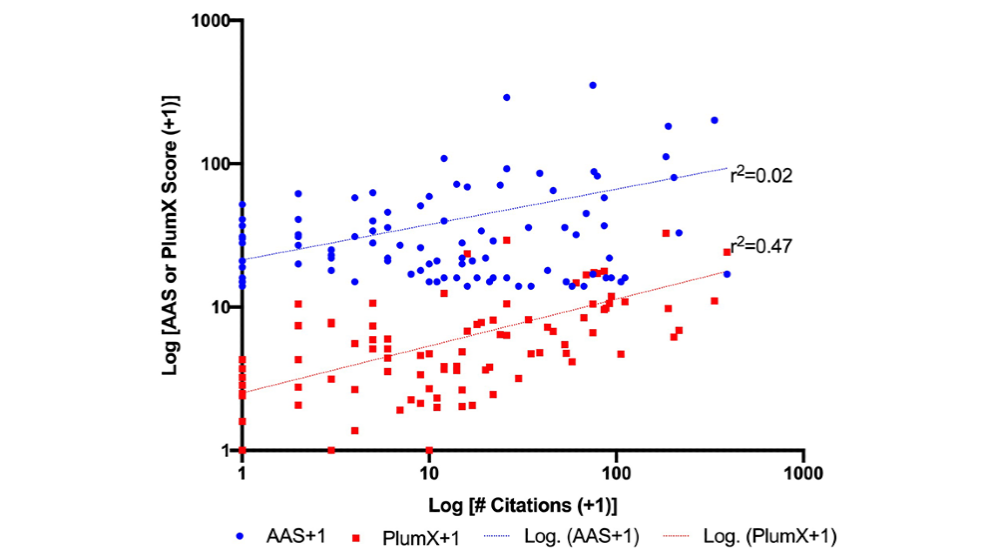
Citation count correlation with the altmetric attention score (AAS) and PlumX score.

**Figure 2:**
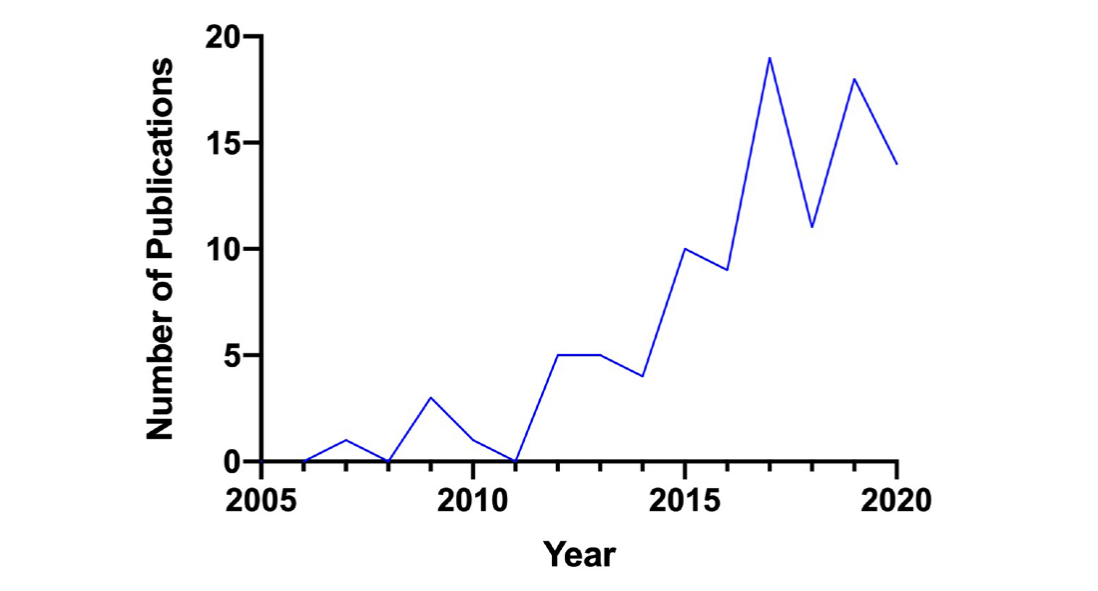
Year of publication for the included articles.

Of the top 100 “trending” hidradenitis suppurativa articles by highest altmetric attention score, most were published in *JAMA Dermatology* (n=27, 27%), followed by *Journal of the American Academy of Dermatology* (n=16, 16%) (Table 1). The median altmetric attention score, PlumX score, and citation count were 25.5, 3.7, and 10.5, respectively. Majority of the “trending” hidradenitis suppurativa research articles were published by authors from Europe (n=47). The median journal impact factor was 6.9. The highest altmetric attention score article was a review article (altmetric attention score=352) [8]. This review article discussed the diagnosis, epidemiology, and treatment of hidradenitis suppurativa, with specific emphasis on advances in the past 5 years [8]. A total of 58 articles had an altmetric attention score above 20, a marker for the top 5% of all scientific output [4]. Of the social media platforms, the most mentions came from Twitter (2830 mentions), which was mostly used to share European-affiliated articles. The second most mentions came from news (573 mentions), which was mostly used to share North American–affiliated articles. The number of Mendeley readers and Dimension citations were 5130 and 4152, respectively. Most of the articles were original articles (53 articles). A total of 49 articles were open access. The most common study design utilized was the clinical observational design (34 articles). A correlation was observed between the altmetric attention score and journal impact factor (*r*^2^=0.17, *P*<.001). Although no correlation was observed between citation count and the altmetric attention score (*r*^2^=0.019, *P*=.17; Figure 1), a positive correlation was observed between citation count and the PlumX score (*r*^2^=0.469, *P*<.001). The altmetric attention score was further found not to be correlated with citation count for particular article types in a chi-square analysis (*P*=.95). Open-access status did not affect the altmetric attention score (*P*=.71). Out of the 100 articles, most of the articles were published in 2017 (19 articles), followed by 18 articles published in 2019 (Figure 2).

**Table 1:** Characteristics of the top 100 “trending” hidradenitis suppurativa articles by the altmetric score.

Characteristic		Value (N=100)
**Journal, n**		
	*JAMA Dermatology*	27
	*Journal of the American Academy of Dermatology*	16
	*British Journal of Dermatology*	5
	*Journal of Investigative Dermatology*	4
	Other journals	48
Altmetric score, median (range)		25.5 (13-352)
PlumX field score, median (range)		3.7 (0.0-31.8)
Journal impact factor, median (range)		6.9 (0.2-70.7)
Traditional citation, median (range)		10.5 (0-389)
News mentions, total (range)		573 (0-109)
Policy mentions, total (range)		17 (0-3)
Twitter mentions, total (range)		2830 (0-706)
Facebook mentions, total (range)		190 (0-24)
Number of Mendeley readers, total (range)		5130 (0-245)
Number of Dimension citations, total (range)		4152 (0-418)
**Article type, n**		
	Original investigation	53
	Research letter/comments to the Editor/brief report	6
	Review	30
	Editorial	4
	Viewpoint/clinical pearls	2
	Guidelines/specific statement	2
	Other	3
	Open access	49
**Study design, n**		
	Case report/series	6
	Clinical observation	34
	Clinical trial	5
	Basic science	7
**Region of the article’s authors, n**		
	Europe	47
	North America	37
	Other	16

## Discussion

### Principal Findings

This study determined the characteristics of the top 100 “trending” hidradenitis suppurativa articles that received the greatest amount of social media attention captured in the altmetric database. Twitter was the most widely used social media platform when mentioning hidradenitis suppurativa. The article with the highest altmetric attention score, a review paper with European affiliation, achieved this high score likely because this article not only summarized the current knowledge about hidradenitis suppurativa, but also went on to provide new insights into the advances in hidradenitis suppurativa research with clear recommendations [8]. Open access, which is often an added cost to authors, did not contribute to a higher altmetric attention score. This likely occurred owing to the decline of the open-access advantage from the ease of article redistribution [9].

### Comparison With Prior Work

Currently, there are no published studies evaluating the utilization of altmetrics with regard to the hidradenitis suppurativa literature. There are however multiple past altmetric studies that investigated top “trending” articles in the altmetric database regarding the correlation between traditional bibliometrics, such as citation count, and online attention, specifically the altmetric attention score. These studies included research articles about specific conditions, such as stroke and oral cancer, and also about specific medical fields, such as urology, emergency medicine, and plastic surgery [10-14]. In our study, altmetrics did not correlate with citation count in hidradenitis suppurativa literature despite its ability to rapidly assess how widely an article is disseminated, while the PlumX score did show correlation. Variable results have been reported regarding the utility of altmetrics [2,4,10-14]. While some articles reported findings similar to those in our study [10,12,13], others only showed a modest correlation at best between citation count and the altmetric attention score [2,4,11,14]. This is likely because the altmetric attention score is more dynamic than the PlumX score since the altmetric attention score focuses on tracking of real-time public interest in a particular topic. Thus, the altmetric attention score does not factor in citation count. In contrast, the PlumX score is likely a better marker because PlumX metrics factor in citation count, suggesting that PlumX metrics may be more useful in identifying high-impact hidradenitis suppurativa articles.

### Limitations

There are several limitations with this study. This study was a cross-sectional study performed in July 2020, so altmetric and PlumX scores may change in the future. Altmetric and PlumX scores should be cautiously interpreted as these alternative metrics do not reflect article quality. Thus, a research article that receives a wide amount of social media attention should not be interpreted as having important results and should be examined in conjunction with in-depth article analysis to determine research quality. Additionally, since the article sample utilized in this study represented a minority of published articles on hidradenitis suppurativa, the generalizability of the findings may be limited regarding hidradenitis suppurativa literature. However, our analysis assessed all hidradenitis suppurativa articles that were in the top 5% of scientific output in the altmetrics database. More recently published hidradenitis suppurativa articles can experience a delay in their citation count compared with older articles. Lastly, our analysis did not assess the presence of journal Twitter accounts or whether the journals were active on Twitter. It is possible that journals having a larger social media presence may bolster their hidradenitis suppurativa articles with higher altmetric scores.

### Future Directions

The correlation of the PlumX score and the altmetric attention score with citation count for other dermatology topics remains to be explored. Further research into identifying specific traits of hidradenitis suppurativa articles with a higher PlumX score or altmetric attention score is warranted. Given the increasing usage of social media by medical professionals and researchers, such research can be useful to investigators by helping them understand the best way to maximize their reach, including the general public. However, as is suggested by our study, it must be understood that the level of research dissemination across social media does not necessarily translate to an impact in the scientific community.

### Conclusion

Since altmetrics and PlumX metrics are dynamic reflections of the general social media interest, there is still some discordance between scientific articles and social media. Although these metrics can identify the impact and dissemination to the public, they do not measure the stringent review process that articles undergo for publication in scientific journals. Despite these limitations, both metrics can be used complementary with traditional citation analysis to assess article quality. Altmetrics with PlumX may be used to rapidly capture what the general public is interested in regarding hidradenitis suppurativa, while traditional metrics can be used to assess an article’s impact.
