# Corrigendum: Assessment of Altmetrics and PlumX Metrics Scoring as Mechanisms to Evaluate the Top 100 Trending Hidradenitis Suppurativa Articles on Social Media: Cross-Sectional Study

**DOI:** 10.2196/106381

**Published:** 2026-08-18

**Authors:** JMIR Publications Editorial Office

**Affiliations:** 1 JMIR Publications Toronto, ON, Canada

Following the publication of “Assessment of Altmetrics and PlumX Metrics Scoring as Mechanisms to Evaluate the Top 100 Trending Hidradenitis Suppurativa Articles on Social Media: Cross-Sectional Study” [1], concerns were raised regarding potential conflicts of interest between reviewers Alex Gu and Vikas Kotha and the authors. These concerns included prior co-publication with members of the author list and active professional relationships between the reviewers and an author. These conflicts were not disclosed to the JMIR Publications Editorial Office as per journal policy.

This article has been subsequently reassessed by the JMIR Publications Editorial Office, who determined that the content of these reviews did not unduly influence the decision to accept the article. The decision to accept the submission was based primarily on the remaining reviewer’s comments and the editor in chief’s contemporary appraisal of the manuscript.

As such, the JMIR Publications Editorial Office issues this corrigendum to update the Conflicts of Interest statement:

Reviewers Alex Gu and Vikas Kotha were found to have undisclosed conflicts of interest with members of the author list under the terms of JMIR Publications policy. The JMIR Publications Editorial Office determined that the content of the reviews did not unduly influence the decision to accept the article. The decision to accept the submission was based primarily on the remaining reviewer’s comments and the editor in chief’s contemporary appraisal of the manuscript.

We regret that these issues were not identified before publication.

The correction will appear in the online version of the paper on the JMIR Publications website together with the publication of this correction notice. Because this was made after submission to PubMed, PubMed Central, and other full-text repositories, the corrected article has also been resubmitted to those repositories.
